# Vibrotactile Coordinated Reset Stimulation for the Treatment of Neurological Diseases: Concepts and Device Specifications

**DOI:** 10.7759/cureus.1535

**Published:** 2017-08-02

**Authors:** Peter A Tass

**Affiliations:** 1 Department of Neurosurgery, Stanford University

**Keywords:** neuromodulation, vibrotactile stimulation, non-invasive stimulation, desynchronization, coordinated reset, parkinson's disease

## Abstract

Coordinated reset stimulation (CRS) consists of spatiotemporal sequences of stimuli delivered to different sites in the brain. Computationally, it was shown that by achieving an unlearning of abnormal synaptic connectivity, CRS can cause a long-lasting reduction of pathological synchronization, a hallmark feature of Parkinson’s disease and other brain disorders. Pre-clinical and proof of concept clinical studies in parkinsonian monkeys and patients showed that CRS applied through deep brain stimulation electrodes implanted in the subthalamic nucleus resulted in cumulative and long-lasting therapeutic effects along with a reduction of beta band oscillations. To apply CRS noninvasively by vibrotactile stimulation delivered to different fingertips, we present three different possible stimulation concepts. These different CRS approaches target different mechanoreceptors and related stimulus mechanisms. The different approaches are based on the diverse physiology of mechanoreceptors and dynamic CRS principles. Required stimulation parameters and specifications provide a guideline for technically implementing vibrotactile CRS during clinical tests.

## Introduction

Abnormal neuronal synchrony may severely impair brain function, for instance, in Parkinson’s disease (PD) [1]. During the 19th century, Charcot observed that after a long carriage train or horseback ride, PD patients experienced marked symptoms of amelioration [2]. This led to attempts to develop vibratory clinical devices for the treatment of PD, which was, however, soon replaced by advanced neuro surgical and pharmacological treatment options [2]. Now, high-frequency (HF) deep brain stimulation (DBS) is the gold standard for the treatment of medically refractory PD [3]. For HF DBS, a train of charge-balanced electrical pulses is permanently delivered at high frequencies (> 100 Hz) to target areas like the thalamic ventralis intermedius (VIM) nucleus or the subthalamic nucleus (STN) via chronically implanted depth electrodes [3].

CRS was developed based on a computational approach targeting the design of stimulation techniques that specifically counteract the abnormal neuronal synchrony by desynchronization [4]. CRS consists of characteristic sequences of brief phase resetting stimuli administered to different subpopulations within an abnormally synchronized neural network [4]. The initial computational studies were performed in neural networks with the fixed and abnormally up-regulated strength of neuronal interactions [4]. Hence, these model networks generated nothing but abnormally synchronized activity, whereas desynchronized states were not stable. Accordingly, the initial intention behind the development of CRS was to restore and maintain desynchronized firing by means of demand-controlled CRS [4]. For this purpose, a demand-controlled timing of stimulus delivery or periodic administration of CRS with demand-controlled stimulus intensity was performed [4].

Spike timing-dependent plasticity (STDP) is a fundamental mechanism of the nervous system that enables neurons to adapt the strength of their synapses to the relative timing of their action potentials [5]. Taking into account STDP in computational model networks opened up a qualitatively new perspective for the development of desynchronizing stimulation protocols [6]. In the presence of STDP, neural networks became plastic; in mathematical terms, it can be stated as ‘multistable’. The networks could attain qualitatively different attractor states. For instance, a network could be synchronously active with strongly up-regulated synaptic connections. Conversely, the network could be in a desynchronized regime with down-regulated synaptic weights. Hence, the research focus moved from a demand-controlled desynchronization [4] to an induction of long-lasting sustained beneficial stimulation effects that outlasts the cessation of properly designed stimulation [6]. From a computational perspective, this was achieved by moving neural networks from pathological model attractor states with abnormally strong synchrony to more physiological model attractor states with down-regulated synchrony [6]. Specifically, in computational studies, it was shown that the CRS-induced desynchronization causes a decrease of the rate of coincidences and, in turn, a decrease of the average synaptic weight, which may ultimately move the network from “pathological” attractors (with abnormally strong synchrony) to more “physiological” attractors (with desynchronized neural activity) [6]. The initial computational studies aimed at the development of novel invasive brain stimulation therapies for movement disorders and epilepsy [4,6]. In a pre-clinical study in parkinsonian nonhuman primates (MPTP monkeys), electrical CRS was delivered through depth electrodes to the subthalamic nucleus (STN) with a daily dose of two hours per day during five consecutive days [7]. Assessments of motor function showed both acute effects and sustained long-lasting therapeutic after effects of CRS-DBS for up to 30 days. In a human proof-of-concept study in six externalized parkinsonian patients, electrical CRS-DBS delivered to the STN on three consecutive days for up to 2x2 hours per day caused a significant and cumulative reduction of STN beta oscillations together with a correlated significant improvement of motor function [8].

Initially, the CRS approach had been developed for invasive brain stimulation, especially DBS [4,6]. Computationally, it was shown that a CRS-induced anti-kindling can also be achieved by means of sensory stimulation [9]. Acoustic CR stimulation was developed to counteract abnormal neuronal synchrony related to chronic subjective tinnitus by means of CRS sound patterns [10]. The tonotopic organization of the central auditory system was employed to enable a separate stimulation of subpopulations. At this point, electrical stimulation bursts were applied to different brain sites for CRS-DBS, which was replaced by acoustically delivering tones of different pitch [10]. A clinical proof-of-concept study demonstrated that therapeutic effects of acoustic CRS achieved after 12 weeks of treatment with a daily dose of four-six hours were significant with respect to baseline and persisted throughout a pre-planned four-week therapy [10]. In addition, electroencephalogram (EEG) recordings demonstrated that the clinical effects of acoustic CRS were combined with a significant decrease of tinnitus-related patterns of abnormal neuronal synchrony [10].

The somatosensory pathway may provide another opportunity to deliver CRS non-invasively, thereby targeting abnormal neuronal synchrony characteristic of movement disorders or epilepsy [1,11]. However, in contrast to the auditory system, there is a variety of different peripheral somatosensory receptors carrying information from muscles, tendons, joints, and skin including four types of cutaneous mechanoreceptors [12-13]. Due to the complexity of the peripheral somatosensory system, there is not just one possible realization of vibrotactile CRS (vCRS) stimulation. Preferably, this paper presents three different concepts for vCRS based on the response characteristics of the selected target cutaneous mechanoreceptors and related thalamic neurons. These concepts differ with respect to intended stimulus mechanism, resulting in stimulus parameter specifications and the design of possible vibrotactile actuators and the corresponding vCRS patterns. The different vCRS concepts are developed based on basic mechanoreceptor physiology as well as CRS principles and are discussed in the context of first clinical tests.

## Technical report

Selection of target cutaneous mechanoreceptors

For illustration and brevity, in this paper, we focus on excessive neuronal synchrony in PD which manifests itself as synchronized oscillatory firing in basal ganglia and exaggerated phase-amplitude coupling (PAC) of beta phase to broadband gamma amplitude in the EEG over sensorimotor cortex [1,11]. To desynchronize a neuronal population, CRS optimally employs phase resetting stimuli delivered to typically three or more separate subpopulations [1,6-7]. Accordingly, the stimulated skin area needs to have a high density of the selected type of mechanoreceptors corresponding to a large area representation in primary somatosensory cortex (S1), and the different stimulation sites should ideally have relatively similar vibrotactile sensitivity. As reflected by the sensory homunculus, the cortical representations of the hand and in particular, the fingers are large compared to that of other parts of the body [14].

Approximately 17,000 mechanoreceptive units innervate the glabrous skin of the human hand [12]. Based on the response to a sustained step indentation, two major categories of mechanoreceptive afferent units have been classified [12]. The majority (56 %) of units are fast adapting (FA) and respond to moving stimuli as well as to the onset and removal of a step stimulus [12]. In contrast, 44% of the units are slowly adapting (SA) and respond with a sustained discharge [12]. In addition, based on the properties of their receptive fields, both categories are classified into two different types [12]. The fast-adapting type I (FA I) units and the slow-adapting type I (SA I) units have small and well-defined fields. In contrast, the receptive fields of the fast-adapting type II (FA II) units and the slow-adapting type II (SA II) are wider and have obscure borders. Fast adapting I units have also been denoted as RA (rapidly adapting), whereas FA II units have been denoted as PC (Pacinian corpuscles) units. The four different types of human cutaneous mechanoreceptors respond optimally to qualitatively different stimuli [12-13,15]. Edge stimuli and stretch stimuli are optimal for SA1 and SA2 mechanoreceptors, respectively. Edge stimuli (SA 1) units often have a rather irregular sustained discharge, whereas SA 2 units discharge in a regular manner, but often display spontaneous discharge in the absence of tactile stimulation. In contrast, vibratory perpendicular sinusoidal skin displacements in the 30 to 60 Hz range are optimal stimuli for FA I units, whereas vibratory stimuli in the 100 to 300 Hz range are optimal stimuli for FA II units. Fast-adapting type I (FA I), especially SA I units have a pronounced edge contour sensitivity and hence their response is stronger when a stimulating contactor surface is not completely contained in the receptive field [12]. Accordingly, to enhance the FA I responses, instead of a flat, spatially homogeneous contactor surface, one could use a contactor surface with a spatially inhomogeneous indentation profile.

Controlled timing by phase entrainment

We developed three different vCRS aiming at eliciting particularly strong responses of only one type of mechanoreceptor units and corresponding thalamic neurons with controlled timing. For this purpose, I employ comparably simple vibratory stimuli which can straightforwardly be generated with standard reliable mechanical stimulation devices such as piezo actuators (PI USA, MA, USA)

CRS aims to modulate the timing pattern of neuronal populations and specifically to cause mutual phase shifts between different stimulated subpopulations [4]. Accordingly, phasic mechanoreceptor discharges with controlled timing with technically easy to realize mechanical stimulators; we select the FA I and/or FA II units as primary target units for the following reasons.

CRS modulates the collective neuronal discharge pattern by delivering phase resetting stimuli to different subpopulations of a synchronized neuronal population at different times to mutually shift the phases of the different stimulated subpopulations [4]. A phase reset can be achieved by means of a periodic pulse train or smooth (e.g. sinusoidal) stimulus train of several periods length by inducing a phase entrainment. Within a few periods of the phase entrainment, the neurons’ phase dynamics (i.e. discharge timing) gets phase locked to the periodic stimulus and hence is reset (restarted) independently of its initial dynamic state as shown computationally in the context of desynchronizing stimulation [16].

There is experimental evidence for phase entrainment effects of vibratory stimuli at the peripheral as well as central level. For instance, median nerve recordings from single afferent mechanoreceptive units demonstrated that FA I and FA II units preferably discharged on the indentation and retraction phase of vibratory stimuli (in their optimal frequency ranges, 5-50 Hz and 100-300 Hz, with amplitudes as low as -12 dB relative to 1 mm peak to peak amplitude and less) administered perpendicularly to the skin [13]. The FA I units produced fewer impulses at the retraction phase than at the indentation phase and the number of retraction-related impulses decreased to zero much earlier when lowering the stimulus amplitude [13]. Johansson and coworkers investigated the relationship between vibratory stimulus and discharge patterns of afferent mechanoreceptive units by calculating the cycles response, i.e. the average number of vibration-evoked impulses per vibration cycle. For FA II units, a cycle response of one is achieved by vibration amplitudes of -30 dB relative to 1 mm peak to peak skin displacement (corresponding to 0.03 mm) and vibration frequencies between 128 Hz and 400 Hz. In contrast, for FA I units, a cycle response of one is obtained at significantly larger vibration amplitudes, e.g. at -12 dB (corresponding to 0.25 mm) and at considerably smaller vibration frequencies, e.g. 32 Hz. Johansson and coworkers [13] did not quantitatively and precisely analyze to which phase of the vibration cycle the afferent mechanoreceptive discharges were locked.

Neurons in the cutaneous core of the human thalamic somatic sensory nucleus [Ventral caudal (Vc)] respond to vibratory stimuli (with static 0.5 mm indentation and 0.1 mm vibration amplitude) quite selectively with a pronounced phase entrainment [15]. The vibratory stimuli used in that study had a static 0.5 mm indentation and a vibration amplitude of 0.1 mm. Responses of human Vc neurons to stimuli that optimally activate the four different mechanoreceptors were analyzed, employing 32 or 64 Hz vibration for FA I units, 128 Hz vibration for FA II, edge stimuli for SA I and skin stretch for SA II units. Seventeen out of 19 neurons had a significantly stronger response to one stimulus as opposed to the other three. Phase entrainment was studied by means of cycle histograms (i.e. distributions of the phase difference between neuronal discharge and stimulus phase) as well as the percentage entrainment (i.e. the maximum percentage of neurons in any continuous half-cycle of the cycle histogram) [15].

Accordingly, simple stimuli and low-frequency (e.g. at 30-64 Hz) or high-frequency vibration (e.g. at 128-400 Hz) added to a constant indentation enabling to optimally cause a phase entrainment of human thalamic Vc neurons and hence provide modulation of thalamic discharge patterns with comparatively high timing precision.

Large cortical representation

The overall goal is to cause a desynchronization of abnormally synchronized neuronal activity in spatially extended neuronal populations, e.g. in the cortex with devices of a limited number, size and contact surface. In this context, it is relevant that the spatial distribution of mechanoreceptors in different regions of the glabrous skin of the human hand varies considerably. The relative densities of innervation of all four types of mechanoreceptive units in the fingertip vs. the rest of the finger vs. the palm are 4.2 vs. 1.6 vs. 1, respectively [12]. The clear majority of mechanoreceptive units in the fingertip are SA I and in particular FA I units with approximately twice as many FA I units as SA I [12]. The FA II and SA II units constitute approximately an eighth of the fingertip mechanoreceptive units [12]. Moreover, the low density of FA II units is relatively uniform from the wrist to the fingertip [12]. In contrast, the density of the FA I units is maximal in the fingertips, strongly drops to the proximal half of the terminal phalanx and undergoes a further but smaller decrease from the bases of the fingers to the palm [12].

In conclusion, to stimulate large numbers of mechanoreceptive units, large corresponding cortical volumes with a limited number of actors and a limited skin contact surface is favorable while stimulating the FA I units of the fingertips. Alternatively, the nearly homogenous density of FA II units might allow sparing the fingertips by targeting FA II units on the dorsal part of the middle phalanx. Sparing the fingertips might enable patients to more comfortably use their fingers during treatment delivery. On the other hand, a possibly more ergonomic alternative might come with a markedly reduced number of mechanoreceptive units and thus smaller brain volume is stimulated.

Spatial selectivity

Different subpopulations engaged in the abnormal neuronal synchronization process should be stimulated separately with no or little spatial overlap [4,7]. In this regard, we should consider that the receptive field size of FA I and FA II units in the glabrous skin of the human hand is markedly different. With a median of 12.6 sq. mm, FA I receptive fields are about 10 times smaller than receptive fields of FA II units [12,17-18]. Since FA I units are predominately located in the fingertips [12] with focal receptive fields [17-18], one could readily anticipate that their corresponding cortical representation areas can be stimulated with no or little spatial overlap (employing preferred FA I vibration parameters given above). In contrast, due to the comparably large receptive fields of FA II units covering, e.g. an entire finger or more [17-18], it seems of major importance to use small peak to peak vibration amplitudes of 0.1 mm or even less, say 0.03 mm, (see above) to avoid spatially widespread activation [13,15].

Based on the physiological and computational findings discussed above, I propose three different vCRS concepts. All three concepts aim at predominately activating one type of mechanoreceptor units, either FA I or FA II.

Concept 1: Burst-like vibrotactile coordinated reset stimulation with high-frequency vibratory bursts

**Figure 1:**
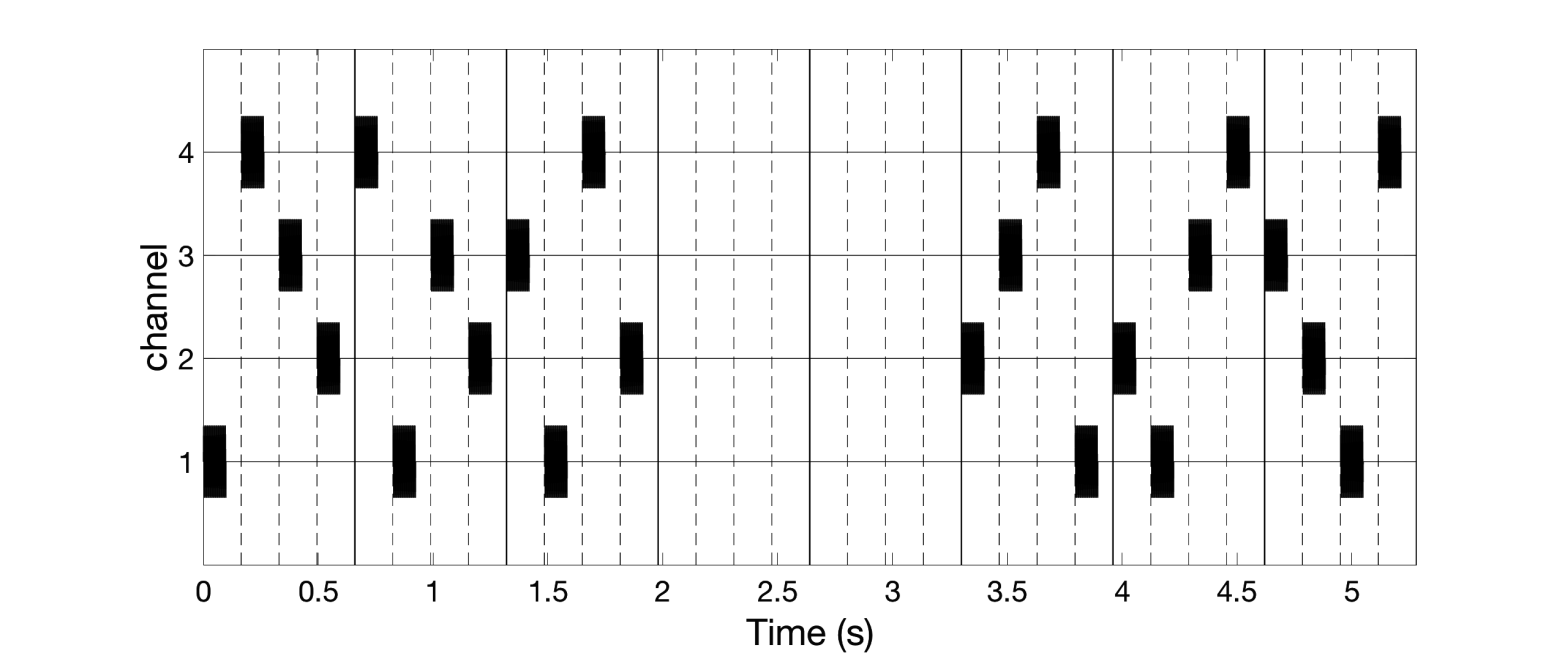
Burst-like vibrotactile coordinated reset stimulation with high-frequency of 250 Hz vibratory bursts (black rectangles) and vibrotactile coordinated reset stimulation period of 660 ms, delivered via four channels. The ordinate is in arbitrary units.

The vCRS frequency differs from the frequency of the vibratory bursts. For instance, the vCRS frequency can be in a low-frequency range such as delta or theta, e.g. 1.51 Hz (Figure 1). The vCRS frequency is defined as vCRS cycle repetition rate. Within one vCRS cycle, one 250 Hz vibratory burst is administered through each channel, respectively. The 250 Hz vibratory bursts are equidistantly spaced in time. Their duration (e.g. equal to 100 ms as in Figure 1) typically does not exceed T/N, where T is the vCRS cycle length and N is the number of channels. Based on computational studies, in other CRS applications, CRS was periodically turned on and off during dedicated cycles to enhance the desynchronizing effect [7,10,19]. Accordingly, Figure 1 shows a vCRS pattern with three cycles on followed by two cycles of stimulation (repeated periodically).

**Figure 2:**
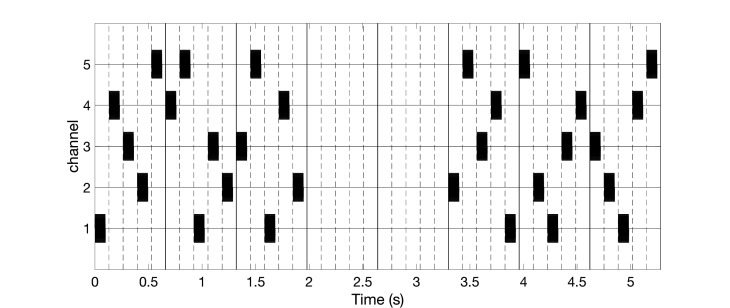
Burst-like vibrotactile coordinated reset stimulation with high-frequency of 250 Hz vibratory bursts (black rectangles) and vibrotactile coordinated reset stimulation period of 660 ms, delivered via five channels. The ordinate is in arbitrary units.

High-frequency vibratory bursts (e.g. at 128-400 Hz) are used to control the timing of the discharges of the FA II units and corresponding thalamic (e.g. Vc) neurons. The vCRS can be delivered via four channels, e.g. to the fingertips of all fingers except for the thumb (Figure 1), ultimately impacting on four different cortical sensorimotor subpopulations. In general, it should typically be three or more channels, e.g. to the fingertips of all fingers of one hand (Figure 2).

**Figure 3:**
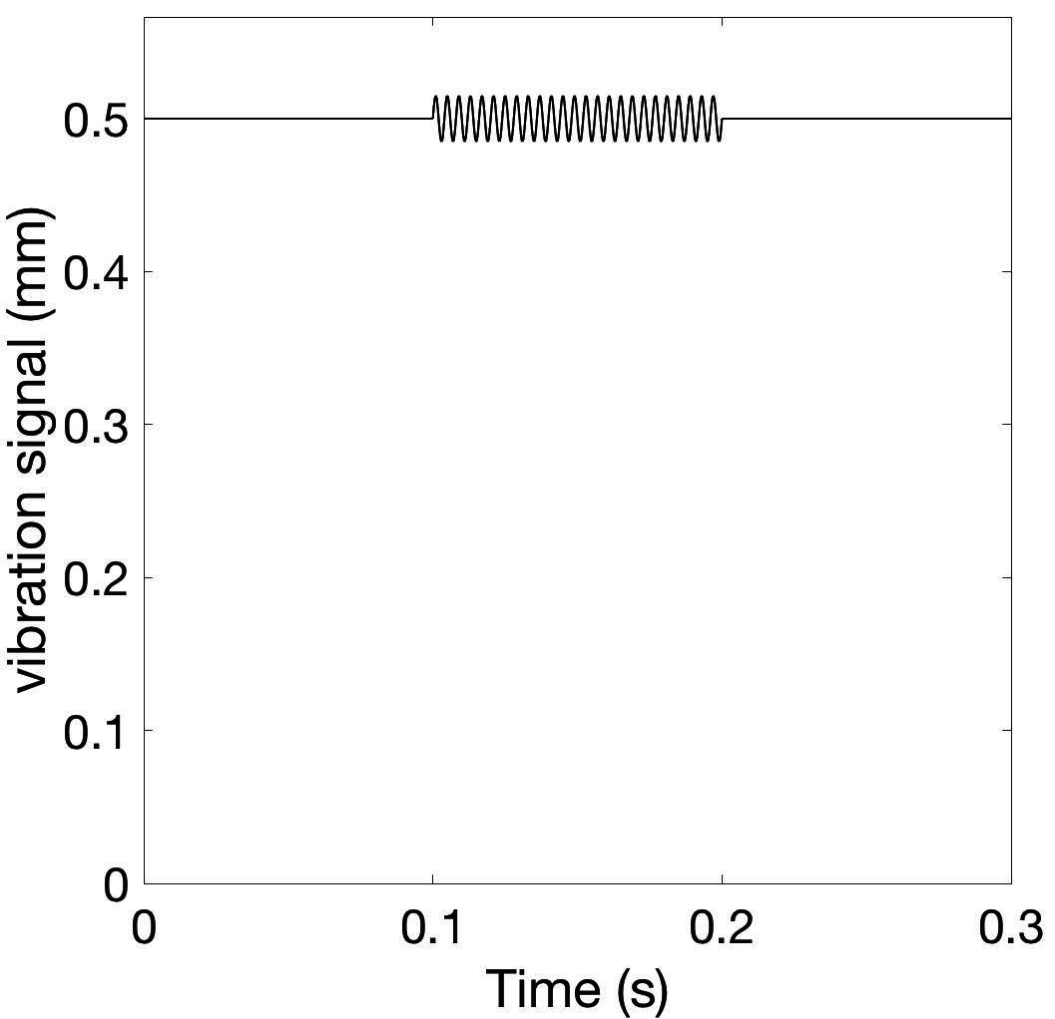
Vibratory burst at 250 Hz. The vibration signal, that is the position of the stimulation contact surface, perpendicular to the skin displays a low-amplitude oscillation (with peak to peak amplitude of 0.03 mm) around the constant indentation of 0.5 mm.

For all channels, the indentation of the stimulation contact surface is constant, e.g. 0.5 mm (Figure 3), throughout the entire vCRS delivery. This can be realized by a permanent fixation of the vibratory stimulation device. The peak to peak amplitude is small, e.g. 0.1 mm or only 0.03 mm (Figure 3). A vCRS sequence is the sequence of channels by which the vibratory bursts are delivered within one vCRS cycle. For instance, the first two vCRS cycles in Figure 1 read 1-4-3-2 and 4-1-3-2. The sequence can randomly vary from one vCRS cycle to the next (Figure 1). Alternatively, the sequence can also undergo slow variations (refer below and discussion section). In Figure 2 the vCRS frequency equals 1.51 Hz = 1/660 ms.

The burst-like vCRS with 250 Hz vibratory bursts at small peak to peak vibration amplitudes of 0.1 mm or even less, e.g. 0.03 mm (Figure 3), aims at predominantly stimulating FA II units and the corresponding thalamic neurons. To stimulate FA II units as selectively as possibly, one should stimulate at particularly low peak to peak amplitudes [13,15]. In addition, to avoid co-stimulation of FA I units, one could stimulate outside of the fingertip, where the density of FA I mechanoreceptors is significantly smaller, e.g. at the dorsal part of the middle phalanx [12].

Concept 2: Burst-like vibrotactile coordinated reset stimulation with low-frequency vibratory bursts

**Figure 4:**
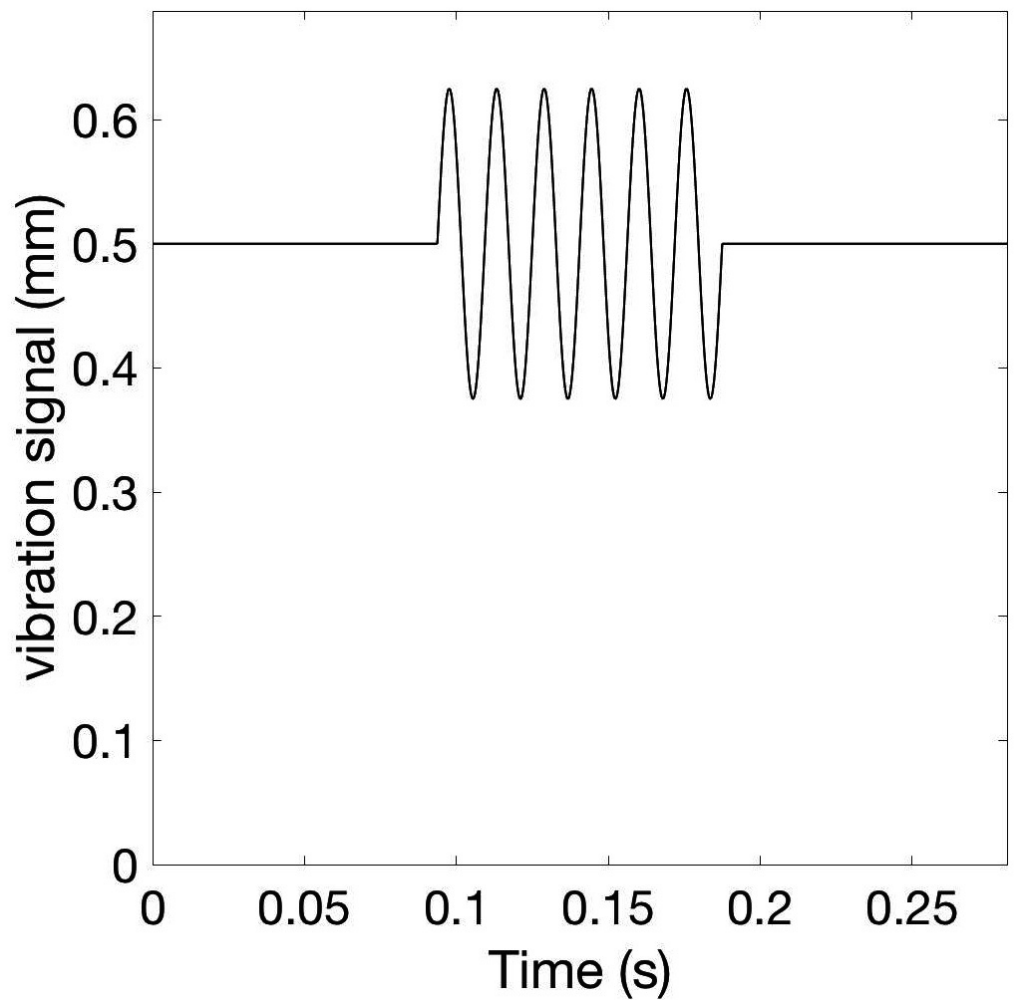
Vibratory burst at 64 Hz. The vibration signal has a peak to peak amplitude of 0.25 mm around the constant indentation of 0.5 mm.

This protocol is similar to the burst-like vCRS with high-frequency vibratory bursts, except for the parameters of the vibratory bursts. To predominately stimulate FA I units and their corresponding thalamic neurons, we employ low-frequency (30-60 Hz) vibratory bursts (Figure 4) that require higher peak to peak amplitudes, e.g. 0.1-0.25 mm. As for the burst-like vCRS with high-frequency vibratory bursts, in this case, we can deliver vCRS via three or four or five channels, i.e. fingertips. This type of stimulation should actually be delivered at the fingertips (as opposed to other parts of the glabrous hand) due to their particularly high spatial density of FA I mechanoreceptors.

**Figure 5:**
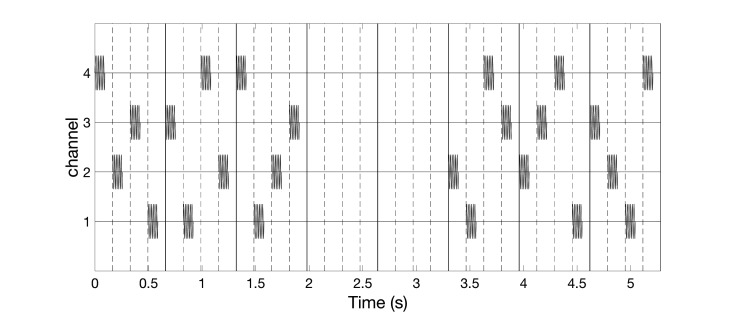
Burst-like vibrotactile coordinated reset stimulation with low-frequency of 64 Hz vibratory bursts and rapidly varying vibrotactile coordinated reset stimulation sequences, delivered via four channels. The ordinate is in arbitrary units.

Burst-like vCRS with both high-frequency (Figure 1) or low-frequency vibratory bursts (Figure 5) can be delivered by randomly varying the vCRS sequence from cycle to cycle. This protocol will be called rapidly varying sequence vCRS. 

**Figure 6:**
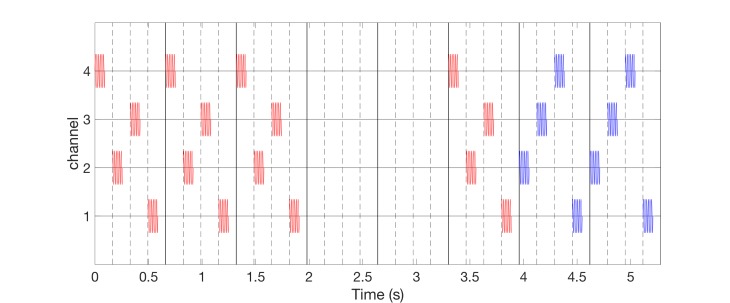
Burst-like vibrotactile coordinated reset stimulation with low-frequency of 64 Hz vibratory bursts and slowly varying vibrotactile coordinated reset stimulation sequences, delivered via four channels. For illustration, random switching occurs after every fourth sequence. Different sequences are indicated by color: First sequence activates channels 4-2-3-1 (red bursts), second sequence channels 2-3-4-1 (blue bursts). Vibrotactile coordinated reset stimulation period is 660 ms. The ordinate is in arbitrary units.

Alternatively, one can also deliver vCRS with slowly varying sequences, where the vCRS sequence is repeated with occasional random switching to the next vCRS sequence. For illustration, in Figure 6, the number of repetitions is four. According to computational studies, the slow variation of CRS sequences may increase the anti-kindling effect [19] (refer discussion section).

**Figure 7:**
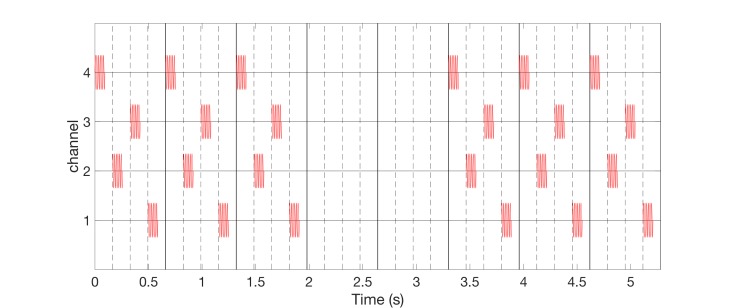
Burst-like vibrotactile coordinated reset stimulation with low-frequency of 64 Hz vibratory bursts and fixed vibrotactile coordinated reset stimulation sequences, delivered via four channels. The fixed vibrotactile coordinated reset stimulation sequence (4-2-3-1) is the same as the first (red) sequence in Figure 6. Vibrotactile coordinated reset stimulation period is 660 ms. The ordinate is in arbitrary units.

Alternatively, one can perform vCRS with fixed vCRS sequence (Figure 7). Burst-like vCRS with both high-frequency or low-frequency vibratory bursts can be delivered via three or more channels (e.g. fingertips). 

Concept 3: Smooth vibrotactile coordinated reset stimulation

**Figure 8:**
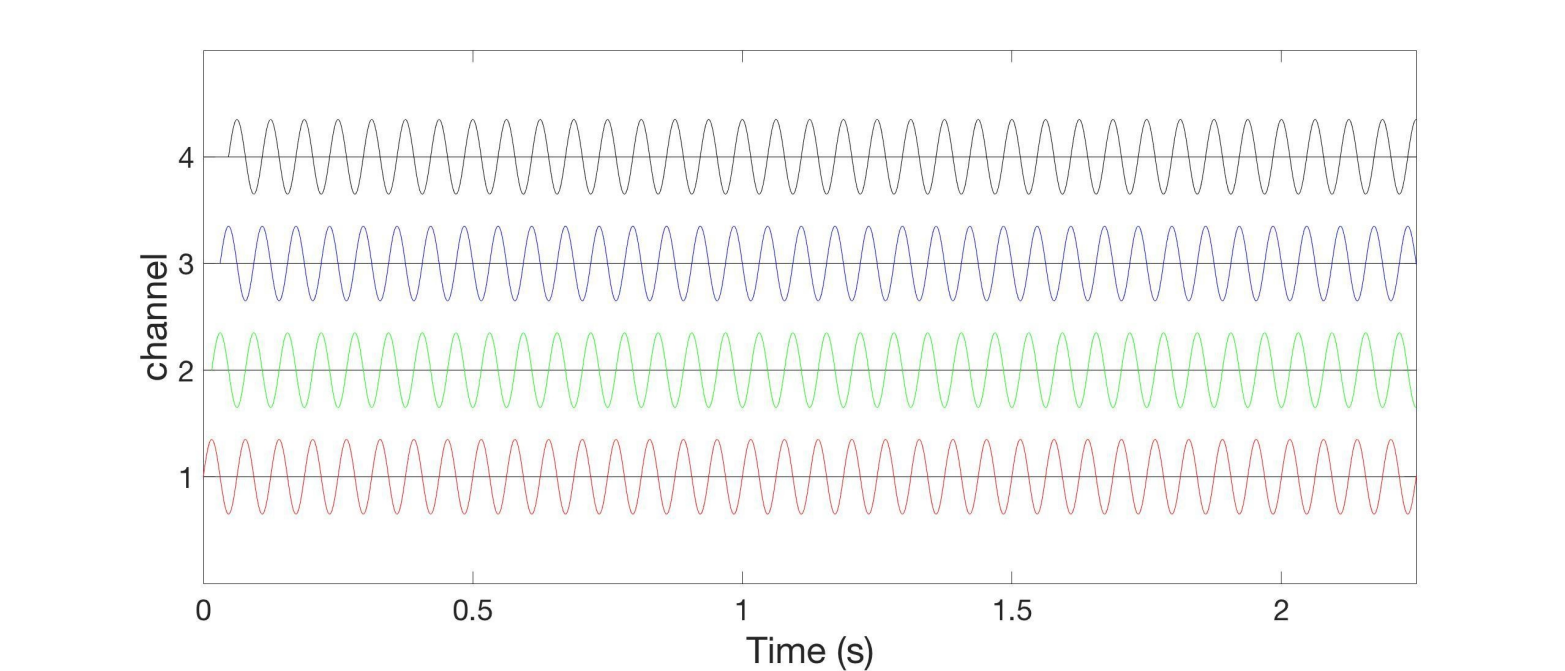
Smooth 16 Hz vibrotactile coordinated reset stimulation with constant phase relationships between different channels without pauses. Colors indicate time shifts of stimulus onset resulting in phase shifts between different channels. Phases of vibratory sine wave stimuli are zero (red), 90° (green), 180° (blue), and 270° (black). Indentation is constant, say 0.5 mm for all channels (not shown). The ordinate is in arbitrary units.

In the two burst-like vCRS protocols discussed above, the vCRS frequency (i.e. vCRS cycle repetition rate) and the (intra-burst) frequency of the vibratory bursts is significantly different. The intra-burst frequency (250 Hz in Figure 1, 64 Hz in Figure 5) is greater than the vCRS frequency (1.51 Hz). Based on the notion of a soft phase rest [16], we now replace a phase resetting vibratory burst by a smooth vibratory train. Accordingly, mutually time-shifted vibratory bursts (as in Figures 1, 3) translate into mutually phase-shifted vibrations (Figure 8). By the same token, in the case of a smooth vCRS stimulation, the vCRS sequence of vibratory bursts corresponds to the pattern of phase shifts between different channels (Figure 8). Accordingly, a burst-like vCRS with fixed sequence (Figure 7) corresponds to smooth vCRS with fixed phase relationships between different channels (Figure 8). 

**Figure 9:**
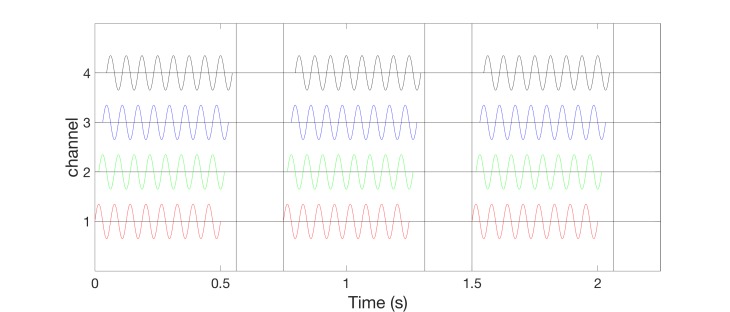
Smooth 16 Hz vibrotactile coordinated reset stimulation with pauses and constant phase relationships between channels. Format as in Figure 8.

Smooth vCRS with constant phase relationship between different channels can be realized by continuously delivering vibratory stimulation without pauses (Figure 8) or with pauses (Figure 9). In Figure 9, a vCRS ON epoch, comprising nine ON cycles with active vibration of at least one channel, that is followed by a vCRS OFF epoch, consisting of four OFF cycles (i.e. a pause). This pattern is repeated periodically. The phase ordering of the four channels is identical in all three vCRS ON epochs.

**Figure 10:**
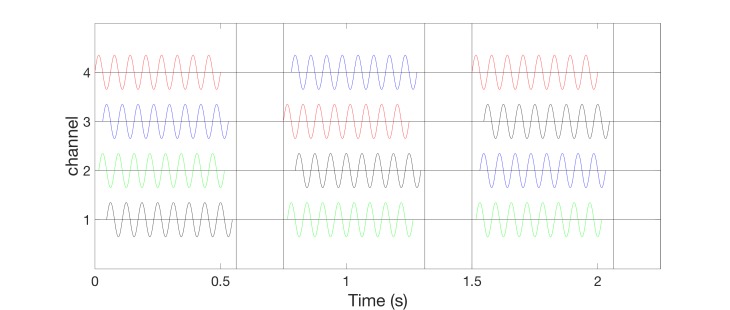
Smooth 16 Hz vibrotactile coordinated reset stimulation with pauses and phase relationships between channels randomly varying after every vibrotactile coordinated reset stimulation ON epoch. Format as in Figure 8.

Alternatively, corresponding to the burst-like vCRS, with slowly varying sequences presented above, in the case of smooth vCRS, the phase relationship may randomly vary from one vCRS ON epoch to the next (Figure 10).

**Figure 11:**
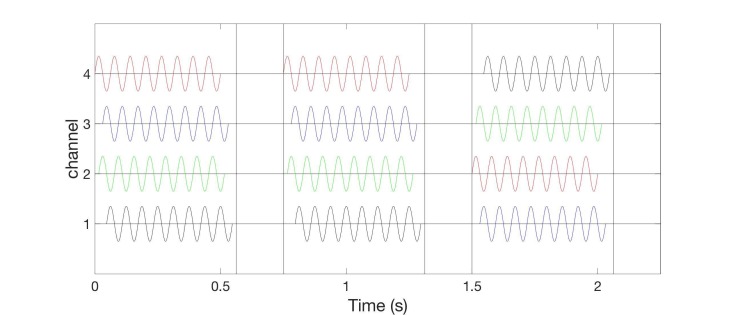
Smooth 16 Hz vibrotactile coordinated reset stimulation with pauses and phase relationships between channels randomly varying after every second vibrotactile coordinated reset stimulation ON epoch. Format as in Figure 8.

The phase relationships between channels can also be randomly varied after every n-th vCRS epoch (n=2 in Figure 11). Smooth vCRS can be delivered through three or more channels (e.g. fingertips). A major difference between the burst-like vCRS and the smooth vCRS protocol is that for burst-like vCRS vibratory stimuli are not simultaneously delivered to different parts of the body (e.g. fingertips).

## Discussion

This paper presents three different vCRS concepts that can directly be implemented technically for clinical studies. The goal of all three concepts is to predominantly stimulate either FA I or FA II mechanoreceptors units and their corresponding thalamic neurons. Due to an intended peripheral and, in particular, thalamic phase entrainment, a relevant population of neurons may produce stimulus-entrained discharges. In contrast, stimulating all four types of mechanoreceptors, SA I, SA II, FA I and FA II might cause stimulus responses with inhomogeneous, compound phasic and tonic timing characteristics. This might lead to less precise timing and, hence, render CRS less effective. In any case, simultaneously all four types of mechanoreceptor units requires time-varying indentation and, hence, large amplitudes requiring sophisticated mechanical stimulation devices. (Tass PA, Mayor L, Roulet J-C, Schnell U: Device for treating a patient with vibration, tactile and/or thermal stimuli. International patent application WO 2011/098082 A1, 1-57, 2011). In contrast, the stimulation concepts presented here can straightforwardly be realized, e.g. by means of standard piezo technology (LGL Group, Inc., Orlando, Florida, United States).

The main advantage of burst-like vCRS at higher intra-burst frequencies, e.g. 250 Hz, and low peak to peak vibration amplitudes may be the selective activation of FA II units [13]. A downside of this approach may be the large receptive field size of FA II units [17-18], which might hinder selective stimulation of separate subpopulations, in particular, in neurological conditions, such as Parkinson’s disease associated with enlarged receptive field size [20]. Stimulating at high amplitudes may activate remote FA II receptors too [17-18]. This might reduce the desynchronizing effect of CRS [7]. Particularly at the large vibration amplitudes, it has undesired, synchronizing effects.

In contrast, burst-like vCRS at lower intra-burst frequencies, e.g. 32-64 Hz, and low peak to peak vibration amplitudes may favorably activate large and separate FA I-related thalamic populations since the density of the FA I units peaks is in the fingertips [12]. Since FA I units require higher vibration amplitudes [13], a co-activation of FA II units might occur. To avoid the latter, employment of lower intra-burst frequencies, say 32 Hz instead of 64 Hz, might be favorable. However, at 32 Hz a vibratory burst contains only half the periods of a 64 Hz burst, which might reduce efficacy since it was shown that both FA I (and FA II) units need a few (less than five) cycles to build up a stable phase entrained stimulus response [13]. This might be compensated by increasing the duration of the vibratory burst and also the number of vibration periods. In addition, one might even reduce the vCRS frequency to allow greater vibratory burst durations.

As an alternative to selectively targeting only FA I or only FA II units, one could also design compound vibratory stimuli and related devices. For instance, one could simultaneously deliver FA I-targeting burst-like 32 Hz vibration to the fingertips in combination with FA II-targeting burst-like 250 Hz vCRS of the dorsal part of the middle phalanx. The vibration frequencies should be commensurate and the vibratory bursts’ indentation or retraction could end coincidently or be adapted to measured propagation delays (see below).

Conduction velocities of FA I units and FA II units are similar. FA I conduction velocities were found to range from 26-91 m/s (with mean 55.3 m/s ± 3.4 m/s) and FA II conduction velocities from 34-61 m/s (46.9 m/s ± 3.6 m/s) (Knibestöl, 1973). Based on computational studies performed so far [4,6,9] for low CRS frequencies (i.e. cycle repetition rates, as in Figures 1 and Figure 3), e.g. fCRS = 1.5 Hz, there is minor variation of the conductance delays of a few ms units belonging to the same as well as to different stimulated sub-populations, which would not be expected to render CRS ineffective. However, for precise calibration, the timing of the vibratory stimuli delivered to the different fingers could be adapted to the individually assessed propagation delays by means of vibration evoked potentials of the different fingertips. Differences of these propagation delays could be compensated by adapting the timing of the onsets of the vibratory stimuli accordingly.

Smooth vCRS may be applied to specifically desynchronize synchronized beta band oscillations. The vibration frequency (16 Hz in Figure 4) could, in principle be adjusted to local field potential recordings from depth electrodes, epicortical electrodes or electroencephalogram (EEG) electrodes [11]. Given its considerably smaller vibration period (as opposed to the cases of burst-like vCRS), propagation delays will likely matter. Imbalances between different channels may hinder efficacy and in extreme cases give rise to multi-channel coincident vibration, potentially causing synchronizing effects. Hence, this approach may be beneficial for the measurement of propagation delays and for the corresponding adaptation of the phase relationships.

Measuring propagation delays might also help to compensate for interhemispheric delays. Based on computational results, for the burst-like vCRS protocols (Figures 1 and 3) one would not expect minor delays to significantly reduce vCRS efficacy. However, this needs to be tested clinically. Furthermore, interhemispheric interference could be avoided by stimulating unilaterally, e.g. by delivering burst-like vCRS to the more affected side. For comparison, in a proof-of-concept study in externalized PD patients, during three stimulation days, CRS, STN DBS was administered unilaterally, which was exclusively contralateral to the more severely affected side [8]. This protocol induced a significant and cumulative reduction of beta band local field potential (LFP) oscillations along with a significant improvement of motor function.

In computational studies, CRS was typically delivered to three or more separate subpopulations of approximately the same size [4,6-7]. Accordingly, it might be favorable, but more involved in adjusting the peak to peak vibration amplitude for each fingertip separately, to equalize stimulus response amplitudes (by EEG) or volumes by functional magnetic resonance imaging (fMRI) and to enable activation of cortical volumes of similar size, thereby compensating for the different size of cortical finger representations [14].

In computational, pre-clinical and clinical studies, CRS was delivered with fixed CRS sequence [4,9], with rapidly varying CRS sequence [4,7-8,10], or with slowly varying CRS sequence [19]. Computationally, it was shown that CRS with slowly varying sequences might cause a more pronounced anti-kindling [19]. However, computationally it was shown that CRS with rapidly varying CRS sequences might be more robust with respect to mutual detuning of CRS frequency and intrinsic neuronal firing/bursting rate (Manos T, Zeitler M, Tass PA: How would be the stimulation frequency and intensity impact on the long-lasting effects of coordinated reset stimulation. To be submitted in approx. two-three weeks). Accordingly, first pilot studies might reasonably employ burst-like vCRS with high-frequency or low-frequency vibratory bursts and rapidly varying vCRS sequences (Figure 1 and Fifure 5). Smooth vCRS should be performed with short vCRS ON epochs comprising a few vibration periods and phase relationships between channels that randomly vary after every vCRS ON epoch. The length of the vCRS ON epoch should be sufficient to induce a phase entrainment. However, it should be insufficient to cause the specific slowly varying sequences effect, requiring 25 or more repetitions [19] of vibration periods with constant phase relationships between channels.”

Apart from delivering vCRS to the fingertips, based on the sensory homunculus and the symptoms under consideration, one could, of course, deliver vCRS stimulation also to other parts of the body. The vCRS might also be tested in other brain disorders characterized by abnormal neuronal synchrony. Possible applications might be thalamocortial dysrhythmia-related diseases, such as neurogenic pain or depression [21].

## Conclusions

The vCRS can technically be realized for clinical tests by means of standard, e.g. piezo technology. Burst-like 250 Hz vCRS at particularly low amplitudes with rapidly varying vCRS sequence may allow for selective activation of FA II mechanoreceptor units and corresponding thalamic neurons. Burst-like vCRS with vibratory bursts at 32-64 Hz and slightly higher peak to peak amplitude and rapidly varying vCRS sequences might be favorable to stimulate large, but separated cortical fingertip representations. A more involved vCRS approach is the smooth vCRS, with phase relationships between channels randomly varying after every vCRS ON epoch. The smooth vCRS approach might require adaptation of the phase relationships to the measured conductance delays.
